# Electroconvulsive Shock Induces Greater Plasticity of Dentate Gyrus Neurons Born in Adulthood Than Those Born in Development

**DOI:** 10.1111/ejn.70492

**Published:** 2026-04-09

**Authors:** T. R. Zhang, B. Askari, Rachel Smith, Likitha Mallela, F. Vila‐Rodriguez, J. S. Snyder

**Affiliations:** ^1^ Department of Psychology University of British Columbia Vancouver Canada; ^2^ Djavad Mowafaghian Centre for Brain Health University of British Columbia Vancouver Canada; ^3^ Non‐Invasive Neurostimulation Therapies Laboratory, Department of Psychiatry, Faculty of Medicine University of British Columbia Vancouver Canada; ^4^ School of Biomedical Engineering University of British Columbia Vancouver Canada

**Keywords:** adult neurogenesis, electroconvulsive shock, hippocampus, plasticity

## Abstract

Electroconvulsive therapy (ECT) is the most effective treatment for severe depression but uptake is hindered due to effects on memory and a poor understanding of its mechanisms of action. Hippocampal plasticity is one consequence of ECT that may be related to both the therapeutic and amnestic effects. In animals, electroconvulsive shock (ECS) dramatically increases adult neurogenesis in the dentate gyrus (DG). However, little is known about how ECS impacts the morphology of adult‐born neurons. Moreover, it is unknown whether ECS differentially impacts DG neurons that are born in development versus adulthood. To address these questions, here, we subjected male and female mice to a clinically relevant schedule of chronic ECS and examined effects on DG neuron populations. As predicted, ECS dramatically increased the survival of adult‐born DG neurons generated shortly before treatment and the number of immature neurons present after treatment. Adult‐born neurons from ECS‐treated mice also had longer dendrites and larger presynaptic terminals, suggesting enhanced circuit integration. In contrast, ECS did not affect the survival of neurons born in early postnatal development and it did not alter the structure of their dendrites or presynaptic terminals. Instead, ECS reduced spine density on developmentally born neurons in the dorsal DG and, in the ventral DG, it increased mushroom spine density. Thus, adult‐born neurons generally display greater ECS‐induced plasticity than developmentally born neurons, which may be relevant for the effects of ECS/ECT on cognition and mood.

## Graphical abstract

Chronic electroconvulsive shock (ECS) increases neurogenesis and promotes the structural growth of adult‐born neurons.

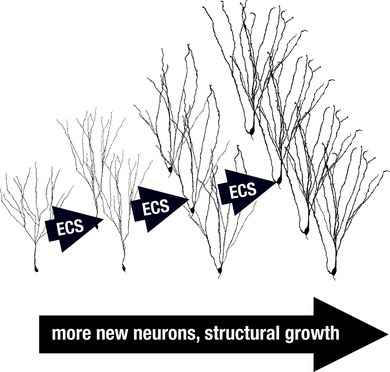

**Abbreviations**
ABN: adult‐born neuron
BrdU: bromodeoxyuridine
DBN: developmentally born neuron
DCX: doublecortin
DG: dentate gyrus
ECS: electroconvulsive shock
ECT: electroconvulsive therapy
MFB: mossy fiber bouton

## 1 Introduction

Electroconvulsive therapy (ECT) is a long‐standing and profoundly effective treatment for mood disorders (Group 2003). While the mechanistic basis of ECT is multifaceted (McClintock et al. 2014), the induction of hippocampal plasticity may be relevant for both its therapeutic and side effects. Indeed, one of the most consistent biological effects of ECT is an increase in hippocampal volume (Gbyl and Videbech 2018; Takamiya et al. 2018), which has been reported to correlate with clinical improvement in some studies (Dukart et al. 2014; Joshi et al. 2016) (but not others (Abbott et al. 2014; Oltedal et al. 2018; Ousdal et al. 2020)) and has also been associated with memory impairments (van Oostrom et al. 2018; Argyelan et al. 2021). Notably, subregional analyses indicate that DG volume increases are particularly robust and are most closely linked to the cognitive deficits and symptom improvement (Takamiya, Nuninga, et al. 2019; Nuninga, Mandl, Boks, et al. 2020; Gbyl et al. 2021). Adult neurogenesis in the hippocampal dentate gyrus (DG) is often cited as potentially important consequence of ECT (Nuninga, Mandl, and Sommer 2020; Tartt et al. 2023). In animals, electroconvulsive shock (ECS) is one of the strongest neurogenic stimuli, reliably increasing the birth of new neurons 2–3× over baseline (Malberg et al. 2000; Perera et al. 2007; Nishida et al. 2008; Yanpallewar et al. 2012; Zhang et al. 2021) and also increasing neuronal survival during the immature critical period (Jonckheere et al. 2018; Ueno et al. 2019). These effects are clinically relevant because neurogenesis reduces depression‐relevant behaviors and hypothalamic–pituitary–adrenal output (Schloesser et al. 2010; Snyder et al. 2011; Surget et al. 2011; Lehmann et al. 2013) and contributes to some of the antidepressant‐like effects of ECS in mice (Schloesser et al. 2015). Furthermore, neurogenesis promotes retrograde amnesia in rodents (Akers et al. 2014), one of the most problematic side effects of ECT in patients (Lisanby et al. 2000).

The patient and preclinical data therefore point to the DG and neurogenesis as relevant targets for understanding the effects of ECS/ECT. And while adult‐born neurons (ABNs) comprise a large proportion of the DG (Snyder and Cameron 2012; DeCarolis et al. 2013; Cole et al. 2020), many DG neurons are born in early development and yet little is known about how they are affected by ECS. Developmentally born DG neurons (DBNs) die off at a modest but consistent rate in young adulthood (Dayer et al. 2003; Cahill et al. 2017; Ciric et al. 2019, 2026), raising the question of whether ECS might also promote their survival or loss. By regulating the addition and loss of cells at different stages of life, ECS could dramatically alter the cellular composition of the DG, which could impact its functions in emotion and cognition.

Beyond the mere addition of new cells, it is also necessary to consider that ECS‐induced neurogenesis adds a type of DG neuron that is functionally distinct from earlier‐born cells. During an immature critical period, ABNs typically display more synaptic plasticity (Snyder et al. 2001; Ge et al. 2007), excitability (Mongiat et al. 2009), experience‐dependent plasticity (Tronel et al. 2010; Bergami et al. 2015; Alvarez et al. 2016), and unique in vivo physiology (Danielson et al. 2016; McHugh et al. 2022; Mugnaini et al. 2023) as compared with mature neurons. Their morphological development is also extended (Overstreet‐Wadiche, Bensen, and Westbrook 2006) and lasts for up to 6 months, or 25% of the lifespan, in rodents (Cole et al. 2020). This pattern suggests that ABNs may be more plastic in response to ECS than DBNs.

To address these gaps, here, we conducted broad and comprehensive analyses of the effects of clinically relevant, chronic ECS in mice. Focusing on the DG in particular, we find that ECS has potent effects on the production and growth of ABNs, while only inducing modest patterns of growth and atrophy on DBNs. Collectively, these data identify novel effects of ECS on hippocampal structure that may be useful for understanding both the therapeutic and off‐target effects of ECT in humans.

## 2 Methods

### 2.1 Animals and General Experimental Design

All procedures were approved by the Animal Care Committee at the University of British Columbia and conducted in accordance with the Canadian Council on Animal Care guidelines. Ascl1^CreERT2^ mice (Kim et al. 2011) were bred with CAG^floxStopTdTomato^ mice (Madisen et al. 2010) to generate offspring that were heterozygous for Ascl1CreERT2 and homozygous for the Cre‐dependent tdTomato reporter, to label newborn neurons for morphological analyses as we have done before (Vyleta and Snyder 2021, 2023; Zhang et al. 2023). A total of 71 mice were used (for ABN labeling: seven male sham, 10 male ECS, 10 female sham, 11 female ECS; for DBN labeling: eight male sham, eight male ECS, nine female sham, eight female ECS). However, not all mice were examined for each measure of neurogenesis; the exact sample sizes for each experiment are provided in the results. Mice had ad libitum access to food and water and were housed on a 12‐h light/dark cycle with lights on at 7 a.m.

**FIGURE 1:**
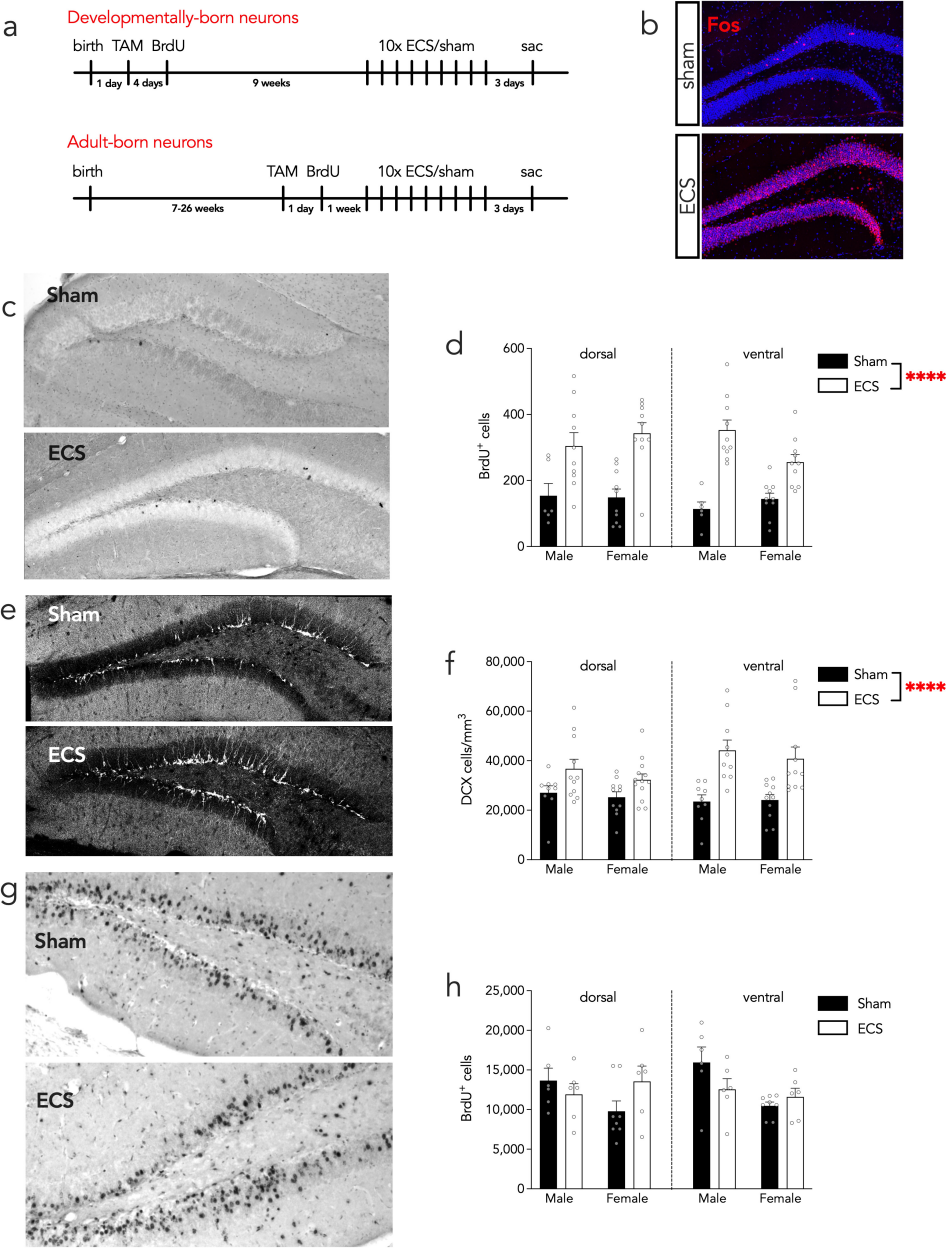
Experimental design and neurogenesis markers following chronic ECS. (A) Experimental timelines. (B) ECS activates the hippocampus, seen as widespread DG Fos expression 1 h after a single ECS treatment. (C) BrdU immunostaining of ABNs. (D) Chronic ECS increased the survival of BrdU^+^ ABNs. (E) DCX immunostaining of immature neurons. (F) Chronic ECS increased DCX^+^ cell density. (G) BrdU immunostaining of DBNs. (H) ECS had no effect on the survival of DBNs. Bars indicate mean ± standard error, and symbols reflect individual animals. ****p < 0.0001.

For DBN labeling, parents were removed from the breeding cage at postnatal day 1 into a separate clean cage while pups were left behind. Pups were injected intraperitoneally with 0.1 mL of 7.5 mg/mL tamoxifen (TAM, Sigma Aldrich) dissolved in sunflower oil to induce whole‐neuronal labeling of newborn neurons (see Figure 1A for experimental timelines). On postnatal day 5, the pups were injected with 50 mg/kg of the thymidine analog, bromodeoxyuridine (BrdU, Toronto Research Chemicals), dissolved in 0.9% saline. Animals were left undisturbed with parents and weaned at 21 days of age.

For ABN labeling, mice were injected with tamoxifen (25 mg/kg in sunflower oil, intraperitoneal) at 7–26 weeks of age. One day later, mice were given one injection of BrdU (200 mg/kg, intraperitoneal). One week after the BrdU injection, all animals were subjected to ECS or sham stimulation. Animals received sham stimulation or ECS every 2 days for a total of 10 sessions over 20 days. The animals were euthanized, and their brains were collected for histological analyses of neurogenesis 3 days following the last stimulation session. Because neurogenesis declines with age, the wide range of ages could potentially confound interpretation of our neurogenesis data if age differed across groups. However, the majority of mice were 7–11 weeks old at the time of ABN labelling, but 1–2 mice in each group were substantially older. Indeed, there were no age differences between the sexes or treatment groups (group means all 73–92 days old at the time of BrdU injection; ANOVA *F*_3,34_ = 0.7, *p* = 0.6).

### 2.2 ECS

One week prior to ECS, animals were handled daily to habituate to handling stress. On the day of stimulation, animals were taken one at a time from their home cage and placed in induction chambers filled with 5% isoflurane with 20% oxygen flow. As soon as animals lost consciousness (could not right themselves), isoflurane was lowered and maintained at 2%. Animals were placed on a wired rack, allowing free limb movement while the body was supported by the rack. Isoflurane at 2% was continuously supplied through a nose cone. A toe pinch was used to test for reflexes. Once lack of reflex was confirmed, the ears were cleaned with 70% isopropyl alcohol and then saline to enhance current conduction. Ear pads were clipped on the ears, and a test conductance loop was run to check for proper electrode placement. A 30‐mA current was delivered at 100 Hz, with 0.5 ms square pulse width, and a total duration of 1 s to trigger a tonic–clonic seizure. If any animal habituated to the stimulation showing no seizure (i.e., increased seizure threshold) at a later stimulation session, the total duration was increased to 1.2 s, and then 1.5 s. No animal required a stimulation duration longer than 1.5 s for seizure induction. Once the seizure had stopped for more than a minute, animals were removed from the setup and placed in a clean recovery cage on a heat pad until they resumed normal behavior. Animals were then returned to their home cage. Sham animals were treated in the same manner, except that after the electrode placement, sham animals were left on the setup with isoflurane for 2 min without any current delivery.

#### 2.2.1 Tissue Preparation and Immunohistochemistry

At the end of testing, mice were transcardially perfused with 4% paraformaldehyde in phosphate‐buffered saline. Brains were bisected to allow one hemisphere to be used for morphological analysis of tdTomato^+^ cells (coronal 100 μm thick sections, cut on a Leica VT1000S vibratome) and the other for histological analysis of neurogenesis markers (coronal 40 μm sections, cut on an AO860 freezing microtome). For Fos immunohistochemistry, brains were cut on the microtome at 40 μm.

BrdU^+^ cells were visualized with DAB immunohistochemistry. Forty micrometer sections were mounted on slides, dried, and 24 h later heated in citric acid at 95°C for 10 min and incubated with trypsin for 10 min for antigen retrieval. Mouse anti‐mouse BrdU antibody (Becton Dickenson) at 1:200 concentration in blocking solution was applied onto the slides for overnight incubation. After washing, slides were incubated with biotinylated goat anti‐mouse secondary antibody, ABC‐HRP kit (Vector), followed by DAB (3,3′‐diaminobenzidine). Lastly, the slides were dehydrated via ethanol and cover‐slipped with Permount.

For free floating fluorescent double immunostaining of doublecortin (DCX), 40 μm brain sections were incubated with rabbit anti‐DCX antibody (1:250 in PBS with 0.1% triton‐x and 5% horse serum) for 3 days on a shaker at 4°C. After the primary antibody incubation, slices were washed and then incubated and stained with donkey anti‐rabbit Alexa 647 antibody (1:250) for 1 h. Fos immunostaining was also conducted on free floating slices, by incubating tissue in 1% H_2_O_2_ for 30 min, rabbit anti‐Fos antibody (1:1000; Synaptic Systems) for 3 days, biotinylated goat anti‐rabbit secondary antibody (Jackson Immunoresearch; 1:250) for 1 h, streptavidin‐HRP for 1 h, and finally streptavidin‐conjugated Alexa 647 (Thermo Fisher). For free‐floating fluorescent immunostaining of tdTomato, 100 μm brain sections were taken from the cryoprotectant solution, washed in PBS, and immunostained with anti‐RFP antibody (1:2000 donkey anti‐RFP, Sigma‐Aldrich) for 1 day followed by rabbit anti‐donkey Alexa 555 (1:250) for 1 h. All slices were stained with DAPI to visualize cell nuclei. Brain slices were mounted onto slides and cover‐slipped with PVA‐DABCO to prevent fluorescent fading.

### 2.3 Imaging and Analyses

All histological imaging and quantification were conducted blind to treatment conditions. All images were acquired with a Leica SP8 confocal microscope. Both dorsal and ventral hippocampal slices were analyzed for all measures. For BrdU and DCX analyses, images were acquired with a 40× oil immersion lens at 1.0 zoom. Images of 1024 × 1024 pixels (400 Hz scan speed) in size at a *z*‐resolution of 1.5 μm were merged to encompass the entire DG. For dendritic analyses, images were acquired at a *z*‐resolution of 1.25 μm with the same 40× oil‐immersion lens at 0.75× zoom. For dendritic protrusions (spines), images were acquired with a glycerol‐immersion 63× objective at 3× zoom, at a *z*‐resolution of 0.5 μm. For bouton and filopodia analysis, images were acquired with the 63× objective at 3× zoom in the CA3a, b, and c subregions at a *z*‐resolution of 0.5 μm.

For BrdU analyses, a one in 12 series of brain sections was analyzed and live‐counted for total positive cells across all DG sections using a Leica brightfield microscope. For DCX histological analyses, four hippocampal sections from each animal were chosen (two dorsal and two ventral sections; dorsal Bregma −1.2 to −2.2 mm, ventral −2.6 to −3.6 mm) and all positive cells were counted on ImageJ using the Cell Counter plugin. Tissue volume was obtained by tracing the two‐dimensional area of the granule cell layer and multiplying this number by the tissue thickness. Positive cell densities (in cubic millimeter) were then obtained by dividing cell counts by the analyzed volume of the granule cell layer.

Neuronal dendrites were traced on ImageJ with the Simple Neurite Tracer plugin (Longair et al. 2011). One to four neurons were taken from each blade (suprapyramidal or infrapyramidal) from either the dorsal or ventral hippocampus. Dendrites were traced three‐dimensionally throughout the *z*‐stack. Dendritic length was calculated as the sum of the lengths of all dendrites for a cell, within a single slice. Branching was defined as the number of dendritic ends. Dendritic protrusions/spines in the outer molecular layer (defined as the outer one‐third of the molecular layer; dendritic segments ~50 μm long) were counted manually on ImageJ with Cell Counter and normalized to the length of the dendritic segment. Typically, two dendritic segments were taken from each blade from both the dorsal and ventral hippocampus, for a total of up to eight dendritic segments counted per animal. Mushroom spines were categorized as having a head diameter of at least 0.6 μm.

For mossy fiber bouton (MFB) analyses, images of bouton terminals were acquired at CA3a, b, and c for both the dorsal and ventral hippocampus (total six images per animal). Five MFBs were analyzed per image (totaling 30 boutons analyzed per animal), with MFBs being identified by their large and irregular shape. MFB cross‐sectional areas were measured on maximum projections of stacked images viewed on ImageJ. Their filopodia, which were identified as protrusions from the MFB that were between 1 and 25 μm in length (Cole et al. 2020), were counted from z‐stacks as they were difficult to visualize on maximum projections of stacked images. The axon terminal boutons and filopodia of newborn DG neurons were analyzed in the CA3 from five animals per sex per stimulation condition. Boutons from the CA3a/b/c subregions were pooled for analyses.

### 2.4 Statistical Analyses

Statistical analyses were performed using R and Prism 10 (GraphPad), and graphs were prepared with Prism 10. Sample sizes for each experiment are indicated in the results section. For BrdU cell counts, results were analyzed by three‐way ANOVA (sex × treatment × dorsoventral subregion). DCX counts were not normal and therefore analyzed by the aligned ranks transformation ANOVA (ART ANOVA with ARTool package in R). Fos cell counts were analyzed by two‐way ANOVA. For morphological analyses, each morphological feature (cell/dendritic segment/bouton) was treated as a separate data point. Morphological data were fit with linear mixed‐effects models (nlme package for R), where sex, treatment, and dorsoventral position were included as fixed effects and subject was included as a random effect to account for correlations resulting from repeated sampling from the same animal (Yu et al. 2021). Fixed effects, including both main effects and interactions, were evaluated using Wald‐type *F*‐tests obtained via the anova() function in nlme. In all analyses, significant effects and interactions were followed up with post hoc Holm–Sidak tests using emmeans. For all analyses, significance was defined as α < 0.05. Underlying data for all graphs are provided as Supporting Information.

## 3 Results

### 3.1 Chronic ECS Increased the Production and Survival of ABNs

To verify that ECS acutely induces widespread activation of the DG, a nonquantitative analysis was performed from tissue from several mice that were given a single sham or ECS session, where brains were collected 90 min later and immunostained for Fos. Whereas sham mice had sparse Fos expression through the DG, ECS‐mice expressed Fos ubiquitously throughout the DG (Figure 1B), consistent with widespread seizure‐induced immediate‐early gene expression (Saffen et al. 1988; Snyder et al. 2009).

Neurogenesis was then evaluated in response to chronic ECS, which significantly increased the number of BrdU^+^ cells born 1 week before treatment, indicating enhanced cell survival in both males and females (Figure 1C,D; sham mice: six males, 10 females; ECS mice: 10 males, 10 females; three‐way ANOVA stimulation effect *F*_1,64_ = 66, *p* < 0.0001; sex effect *F*_1,64_ = 0.3, *p* = 0.6; subregion effect *F*_1,64_ = 0.8, *p* = 0.4; all interactions *p* > 0.05). Chronic ECS also increased the density of DCX+ cells present after treatment, in both sexes (Figure 1E,F; sham mice: nine males, 11 females; ECS mice: 10 males, 11 females; stimulation effect *F*_1,79_ = 28, *p* < 0.0001; sex effect *F*_1,79_ = 3.2, *p* = 0.07; subregion effect *F*_1,79_ = 1, *p* = 0.3; all interactions *p* ≥ 0.06). Because DCX is expressed in cells ranging from hours to weeks old (Brown et al. 2003; Kronenberg et al. 2003; Snyder et al. 2009), this reflects enhanced proliferation and possibly also early survival of immature ABNs born during and after ECS.

### 3.2 ECS Did Not Alter Developmentally Born Neuron Survival

BrdU was injected in pups at postnatal day 5 to test whether ECS alters the long‐term survival of DG neurons born during development (sham mice: six males, eight females; ECS mice: six males, six females). We observed no effects of stimulation on the numbers of neurons (Figure 1G,H; sex effect *F*_1,44_ = 5.5, *p* = 0.02; stimulation effect *F*_1,44_ = 0.07, *p* = 0.8; subregion effect *F*_1,44_ = 0.2, *p* = 0.7; stimulation × sex interaction *F*_1,44_ = 6.4, *p* = 0.015; all other interactions *p* > 0.3). The post hoc comparison was not significant for sham male versus ECS male (*p* = 0.1) or sham female versus ECS female (*p* = 0.8). We found fewer DBNs in females than males in sham animals (post hoc sham female versus sham male *p* = 0.006). This could be due to decreased survival, fewer granule cells in females, or decreased proliferation at the time of labeling due to sex differences in brain development. To ensure that our results were not affected by how we defined dorsoventral boundaries, and because previous studies of DBN survival have examined total cell counts (Dayer et al. 2003; Cahill et al. 2017; Ciric et al. 2019), we also analyzed total stereological cell counts by two‐way ANOVA without subregion as a factor and again found that ECS did not alter DBN survival (stimulation effect, *p* = 1; stimulation × sex effect, *p* = 0.01; post hoc comparisons between sham and ECS treated mice both *p* > 0.1).

### 3.3 ECS‐Induced Dendrite and Spine Growth in ABNs

**FIGURE 2:**
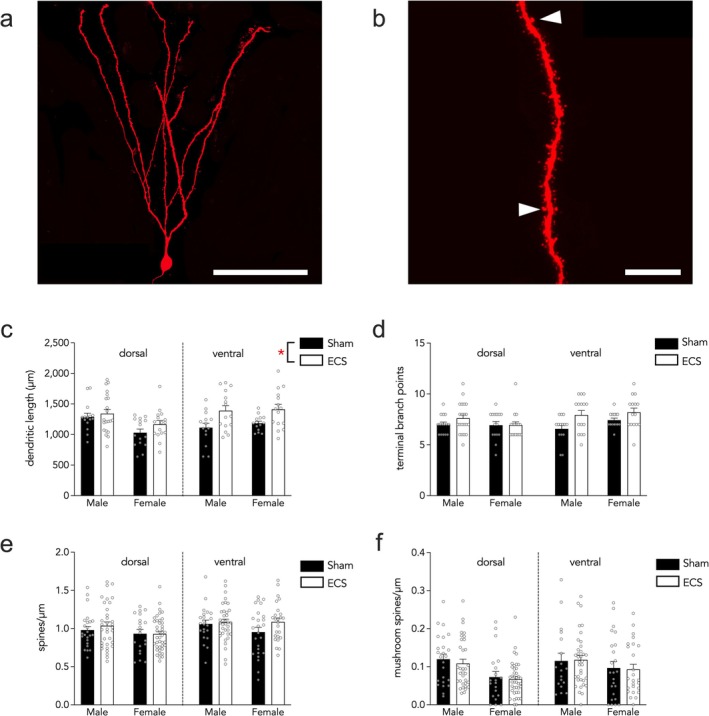
In ABNs, ECS induces dendrite but not spine growth. (A) Confocal image of an ABN. Scale bar, 100 μm. (B) Confocal image of a dendritic segment. Arrows indicate mushroom spines. Scale bar, 10 μm. (C) ECS increased the total dendritic length of ABNs. (D) ECS did not affect branching of ABNs. (E) The spine density of ABNs was not affected by ECS. (F) There was no effect of ECS on mushroom spine density. Bars indicate mean ± standard error, and symbols reflect individual cells. *p < 0.05.

To determine whether ECS alters the morphometric properties of immature ABNs, we first examined the dendritic length of neurons born 1 week prior to ECS (1–6 cells analyzed per mouse, 4–9 mice examined per treatment‐sex‐dorsal/ventral group, for a total of 14–21 cells analyzed per group; Figure 2A). We found that stimulation increased dendritic growth of ABNs. This effect appeared more pronounced in the ventral DG though regional effects of ECS were nonsignificant (Figure 2C; stimulation effect *F*_1,24_ = 5.9, *p* = 0.02; sex effect *F*_1,24_ = 1.5, *p* = 0.2; subregion effect *F*_1,93_ = 6.0, *p* = 0.02; sex × subregion interaction effect *F*_1,72_ = 5.3, *p* = 0.02; all other interactions *p* > 0.1; no significant post hoc comparison for the sex × subregion interaction). The number of terminal dendritic branches was numerically greater in ECS mice though this was not statistically different from sham mice (Figure 2D; stimulation effect *F*_1,24_ = 4.0, *p* = 0.06; sex effect *F*_1,24_ = 0.009, *p* = 0.9; subregion effect *F*_1,93_ = 4.1, *p* = 0.046; all interactions *p* > 0.1).

We next quantified both total spine protrusions on ABNs as well as large mushroom spines, as morphological proxies for input synapses (1–11 cells analyzed per mouse, 5–9 mice examined per treatment‐sex‐dorsal/ventral group, for a total of 20–41 cells analyzed per group; Figure 2B). For overall spine density, there was no effect of ECS or sex on the density of spines (Figure 2E; stimulation effect *F*_1,24_ = 0.18, *p* = 0.7; sex effect *F*_1,24_ = 0.5, *p* = 0.5; subregion effect *F*_1,190_ = 4.1, *p* = 0.04; all interactions *p* > 0.2; no significant post hoc comparison). When we limited our analysis to large mushroom spines, we also did not see any effect of stimulation or sex (Figure 2F; stimulation effect *F*_1,23_ = 0.2, *p* = 0.6; sex effect *F*_1,23_ = 2.3, *p* = 0.1; subregion effect *F*_1,190_ = 2.0, *p* = 0.16; sex × subregion interaction *F*_1,190_ = 4.1, *p* = 0.04; all other interactions *p* > 0.1; no significant post hoc comparisons). There was a significant correlation between overall spine density and mushroom spine density (Pearson's correlation of all animals pooled *r* = 0.589, correlation of ECS animals *r* = 0.65, correlation of sham animals *r* = 0.53, *p* < 0.001 for all correlations).

### 3.4 ECS Increases the Size of ABN Mossy Fiber Terminals

**FIGURE 3:**
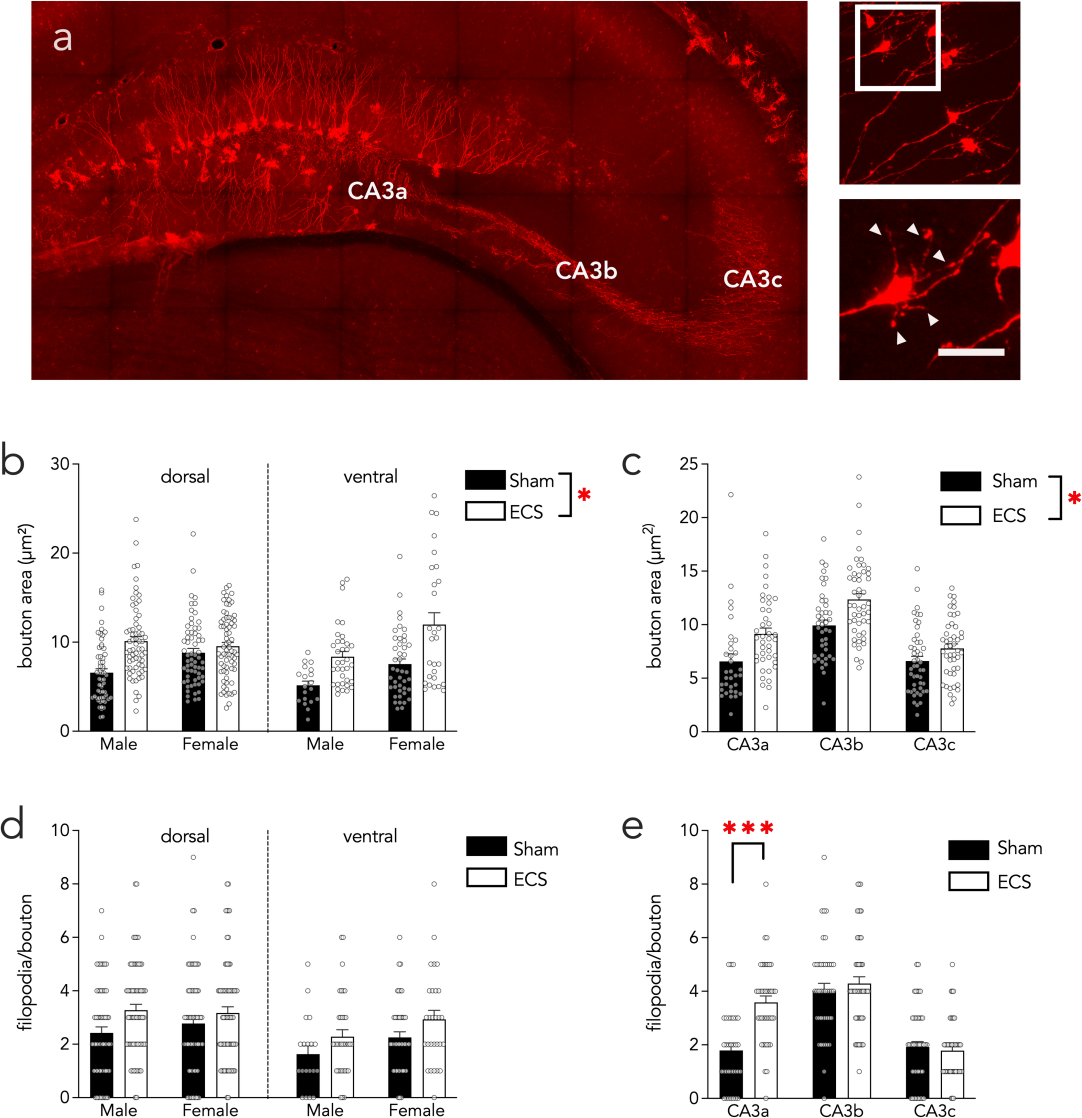
In ABNs, ECS increases the size of presynaptic mossy fiber terminals. (A) tdTomato^+^ ABN neurons in the dentate gyrus, with axons and boutons in CA3. Insets show representative examples of boutons and filopodial protrusions (arrowheads), taken from a different slice than the one shown here. Scale bar, 10 μm. (B) ECS increased the size of ABN presynaptic boutons. (C) In dorsal CA3, ECS similarly induced growth along the proximodistal axis. (D) ECS did not significantly increase the number of mossy fiber filopodia. (E) In the dorsal CA3, mossy fiber boutons in CA3a had more filopodia in ECS‐treated mice. Bars indicate mean ± standard error; symbols reflect individual boutons. *p < 0.05; ***p < 0.001.

We analyzed the axon terminals of newborn boutons, the large MFBs that form detonating synapses onto CA3 cells (3–19 boutons analyzed per mouse, 3–6 mice examined per treatment‐sex‐dorsal/ventral group, for a total of 19–70 boutons analyzed per group; Figure 3A). Mossy fiber boutons were larger in ECS‐treated male and female mice (Figure 3B; stimulation effect *F*_1,18_ = 7.7, *p* = 0.01; sex effect *F*_1,18_ = 1.6, *p* = 0.2). While bouton growth appeared most prominent in the ventral DG, and in the dorsal DG of males, there were no statistically significant regional effects of ECS (subregion effect *F*_1,350_ = 0.9, *p* = 0.3; stim × subregion interaction *F*_1,350_ = 5.0, *p* = 0.03, dorsal sham versus ECS *p* = 0.06, ventral sham versus ECS *p* = 0.08; all other interactions *p* > 0.1). There were differences in bouton size across the CA3 proximo‐distal axis (CA3a/b/c of dorsal hippocampus) but ECS‐induced bouton growth was not specific to any CA3 subregion over another (Figure 3C; LME of dorsal hippocampus CA3 subregion effect *F*_1,224_ = 34, *p* < 0.0001; CA3 subregion × stimulation interaction effect *F*_2,224_ = 1.6, *p* = 0.2).

Filopodia protrusions from the MFB contact local inhibitory neurons to refine the synaptic output of the DG. We did not observe any overall changes in filopodia number after ECS in either sex (Figure 3D; LME model stimulation effect *F*_1,18_ = 3.8, *p* = 0.07; sex effect *F*_1,18_ = 0.6, *p* = 0.5; subregion effect *F*_1,350_ = 10.5, *p* = 0.001; all interactions *p* > 0.3). The number of filopodia per bouton was higher in the dorsal hippocampus compared with the ventral hippocampus. Similar to mossy bouton area, filopodia number varies along the CA3 proximo‐distal axis, with the greatest numbers in CA3b. In our subregion analysis (sexes pooled), there was a significant interaction between CA3 subregion and stimulation; ECS increased filopodia number only in the CA3a subregion but not CA3b or CA3c (Figure 3E; dorsal hippocampus CA3 subregion effect *F*_2,224_ = 51, *p* < 0.0001; CA3 subregion × stimulation interaction *F*_2,224_ = 11.102, *p* < 0.001; post hoc CA3a sham vs. CA3a ECS *p* = 0.0009).

Filopodia number and bouton area were significantly correlated in the overall samples, in ECS animals only, and in sham animals only (Pearson's correlation of all animals pooled *r* = 0.56; correlation of ECS animals *r* = 0.52; correlation of sham animals *r* = 0.55; *p* < 0.001 for all correlations).

### 3.5 ECS Changes Spine, But Not Dendrite, Morphology of Developmentally Born Neurons

**FIGURE 4:**
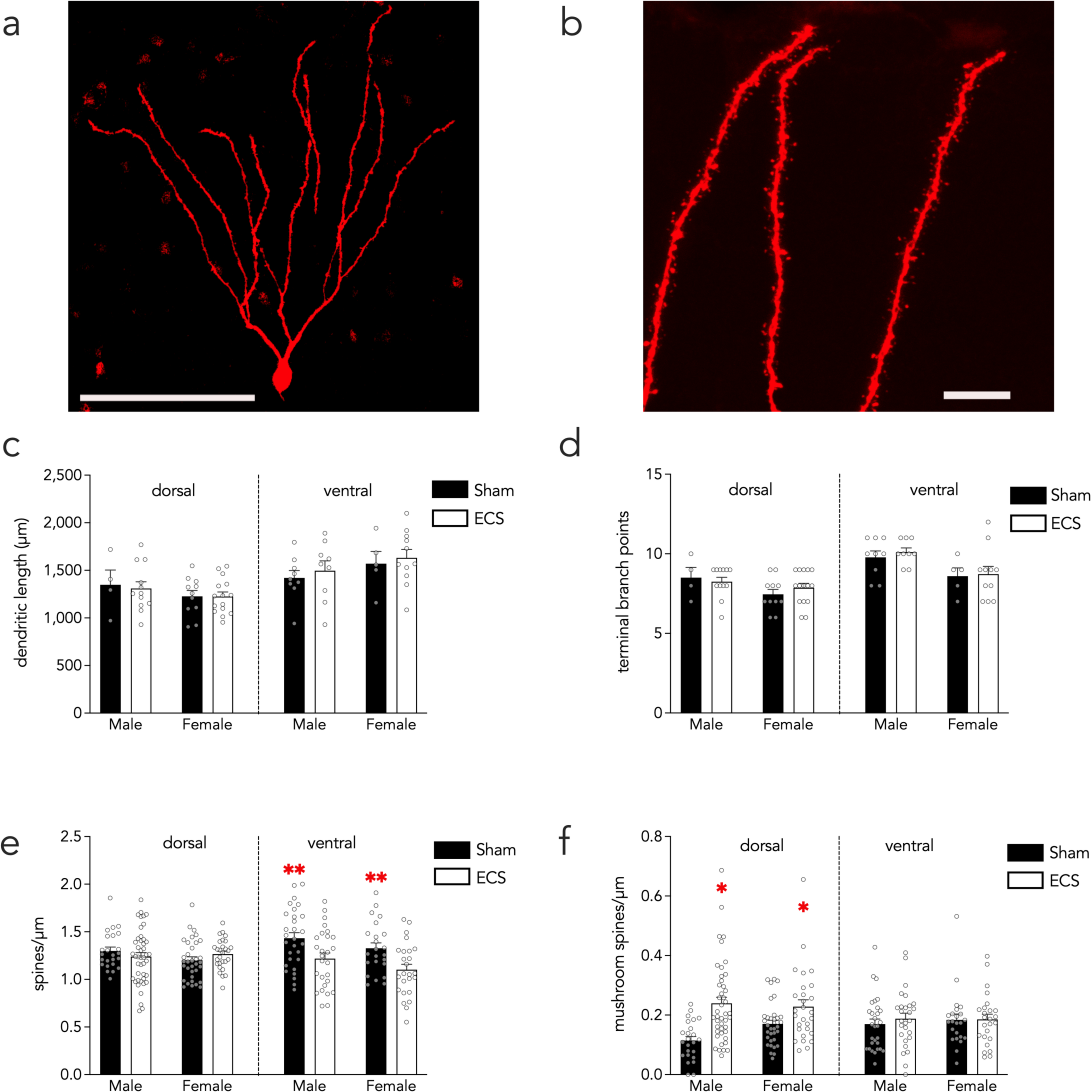
In DBNs, ECS does not alter dendrites but induces bidirectional effects on spine density. (A) Confocal image of a DBN. Scale bar, 100 μm. (B) Confocal image of a dendritic segment. Scale bar, 10 μm. (C) No effect of ECS on the dendritic length of DBNs. (D) ECS did not significantly affect branching of DBNs. (E) ECS decreased the overall spine density of DBNs in the ventral hippocampus. (F) ECS increased the mushroom density of dorsal DBNs. Symbols indicate individual neurons or dendrite segments; bars indicate mean ± standard error. *p < 0.05 dorsal ECS versus dorsal sham; **p < 0.01 ventral ECS versus ventral sham.

We next examined the effects of chronic ECS on the dendritic length of DBNs (1–4 cells analyzed per mouse, 2–6 mice examined per treatment‐sex‐dorsal/ventral group, for a total of 4–15 cells analyzed per group; Figure 4A). DBNs in the ventral DG had longer dendritic length than those in the dorsal DG but there was no effect of ECS (Figure 4C; stimulation effect *F*_1,15_ = 0.3, *p* = 0.6; sex effect *F*_1,15_ = 0.1, *p* = 0.7; subregion effect *F*_1,15_ = 22, *p* < 0.0001; sex × subregion interaction *F*_1,53_ = 4.0, *p* = 0.05, all other interactions *p* > 0.5). Dendritic branching of DBNs was also not affected by chronic ECS, though male mice had more branches than females and dorsal neurons had fewer branches than ventral neurons. (Figure 4D; LME stimulation effect *F*_1,15_ = 0.2, *p* = 0.7; sex effect *F*_1,15_ = 8.7, *p* = 0.01; subregion effect *F*_1,53_ = 26, *p* < 0.0001; sex × subregion interaction *F*_1,53_ = 3.1, *p* = 0.08, all other interactions *p* > 0.5).

ECS bidirectionally affected spine density on DBNs depending on both spine type and dorsoventral subregion (2–10 cells analyzed per mouse, 4–8 mice examined per treatment‐sex‐dorsal/ventral group, for a total of 23–44 cells analyzed per group; Figure 4B). ECS decreased DBN spine density in the ventral DG (Figure 4E; stimulation effect *F*_1,20_ = 3.0, *p* = 0.1; sex effect *F*_1,20_ = 1.4, *p* = 0.3; subregion effect *F*_1,206_ = 0.05, *p* = 0.8; stimulation × subregion interaction *F*_1,206_ = 16, *p* = 0.0001; all other interactions *p* > 0.3; dorsal sham vs. ECS *p* = 0.9, ventral sham vs. ECS *p* = 0.002). There was also a stimulation × subregion interaction for the mushroom spine analysis, where ECS increased mushroom spines only in the dorsal DG (Figure 4F; stimulation effect *F*_1,20_ = 1.9, *p* = 0.2; sex effect *F*_1,20_ = 0.003, *p* = 0.96; subregion effect *F*_1,206_ = 1.1, p = 0.3; stimulation × subregion interaction *F*_1,206_ = 12, *p* = 0.0008; all other interactions *p* > 0.2; dorsal sham vs. ECS *p* = 0.046, ventral sham vs. ECS *p* = 0.9).

### 3.6 Developmentally Born Neuron Presynaptic Boutons Do Not Undergo ECS‐Induced Structural Plasticity

**FIGURE 5:**
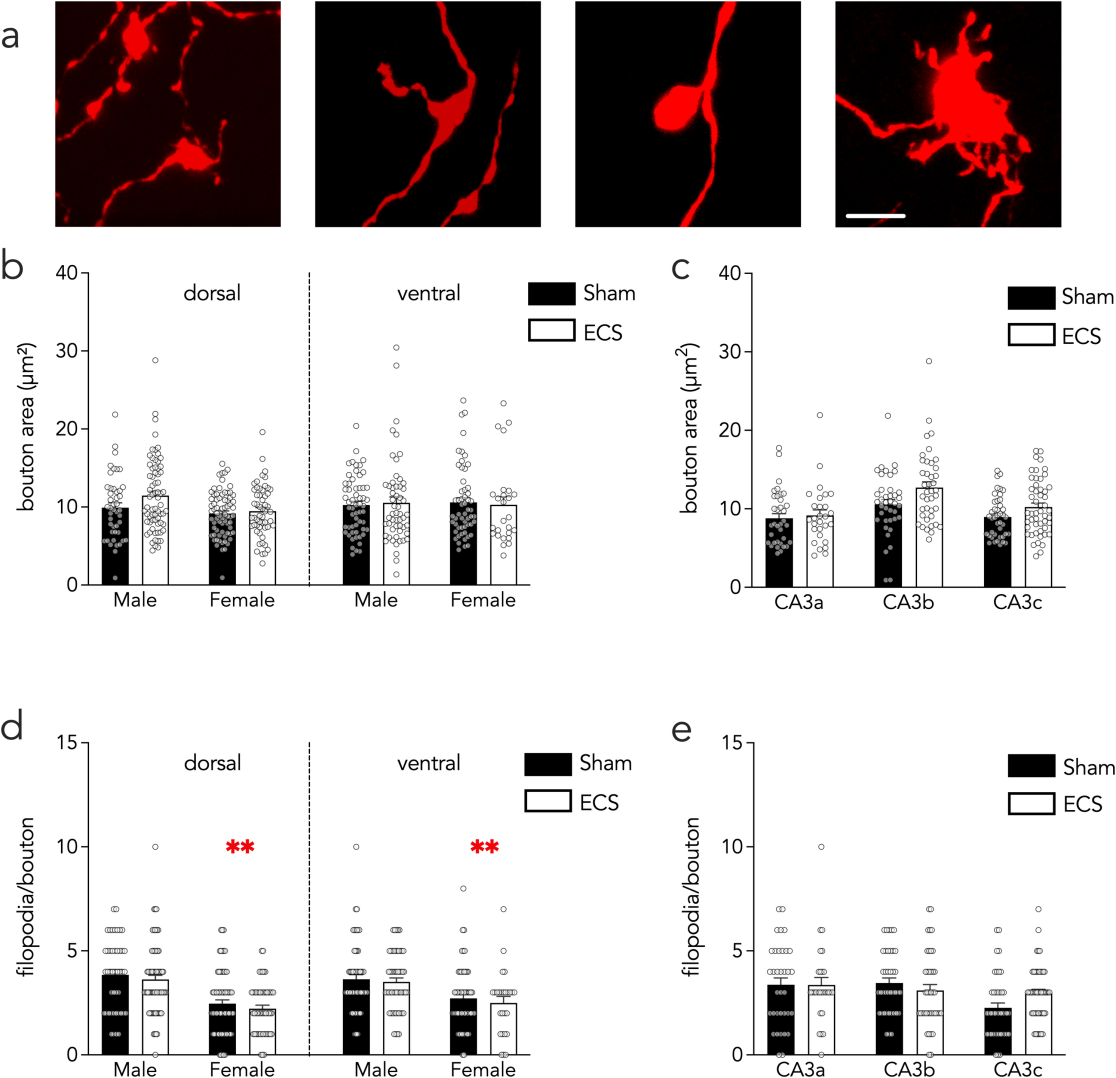
In DBNs, ECS does not alter the morphology of presynaptic mossy fiber terminals. (A) Representative DBN mossy fiber terminals. Scale bar, 5 μm. (B,C) ECS did not affect the size of mossy fiber boutons. (D,E) There was no effect of stimulation on mossy fiber filopodia, but female mice had fewer filopodia per bouton than male mice. Symbols indicate individual mossy fiber boutons; bars indicate mean ± standard error. **p < 0.01 versus filopodia from male mice.

In contrast to the ECS‐induced growth of mossy fiber terminals of ABNs, ECS did not alter the size of DBN terminals (1–17 boutons analyzed per mouse, 3–7 mice examined per treatment‐sex‐dorsal/ventral group, for a total of 26–71 boutons analyzed per group; Figure 5A). There was no effect of stimulation, sex, or dorsoventral subregion on bouton size (Figure 5B; stimulation effect *F*_1,21_ = 0.2, *p* = 0.6; sex effect *F*_1,21_ = 1.2, *p* = 0.3; subregion effect *F*_1,405_ = 0.3, *p* = 0.6; sex × subregion interaction *F*_1,405_ = 5.2, *p* = 0.02, no significant post hoc comparison; all other interactions *p* > 0.1). Bouton size and modulation by ECS also did not vary along the proximo‐distal axis of CA3 (Figure 5C; dorsal hippocampus CA3 subregion effect *F*_1, 217_ = 11.7, *p* < 0.0001; CA3 subregion × stimulation interaction effect *F*_1,207_ = 1.1, *p* = 0.3; all other interactions *p* > 0.3).

We also examined the interneuron‐targeting bouton‐associated filopodia of DBNs (Acsády et al. 1998). Boutons from female mice possessed fewer filopodia than those from male mice, but there was no difference between sham‐ and ECS‐mice (Figure 5D; stimulation effect *F*_1,21_ = 0.003, *p* = 1.0; sex effect *F*_1,21_ = 13.9, *p* = 0.001; subregion effect *F*_1,405_ = 0.4, *p* = 0.5; all interactions *p* > 0.1). Bouton‐associated filopodia did not vary along the proximodistal axis of dorsal CA3 (Figure 5E; LME model subregion effect *F*_1, 207_ = 3.9, *p* = 0.02; CA3 subregion × stimulation interaction effect *F*_2,207_ = 2.4, *p* = 0.09; CA3 subregion × sex interaction effect *F*_2,207_ = 4.1, *p* = 0.02; all other interactions *p* > 0.3). Exploring the CA3 subregion by sex interaction we found that male DBNs had more filopodia than female DBNs in CA3a and CA3b (CA3a female vs. CA3a male *p* = 0.02, CA3b female vs. CA3b male *p* = 0.02; CA3c female vs. CA3c male *p* = 0.8).

## 4 Discussion

ECT has gross effects on hippocampal/DG structure that are difficult to further probe in the human brain. Here, we used a mouse model to identify how ECS impacts the number and morphology of DG neurons born in development versus adulthood. We found that chronic ECS robustly increased the survival of ABNs and expanded the immature neuron pool in both male and female mice. In contrast, there were no changes in DBN cell numbers in mice treated with ECS. At the structural level, ABNs were generally more responsive to the trophic effects of ECS, displaying increased dendritic length and larger presynaptic mossy fiber terminals (neither of which were altered in DBNs). However, chronic ECS did decrease DBN spine density in the ventral hippocampus and it increased DBN mushroom spine density in the dorsal hippocampus. All effects of ECS were equivalent in males and females. Notably, in a separate study of the same tissue, we recently found that the survival and morphology of adult‐born striatal neurons are not affected by ECS (Gaertner et al. 2025). Thus, neurogenic effects of ECS may be greater in the hippocampus than in other brain regions.

### 4.1 Hippocampal Volume Changes and the Survival of Neurons Born in Development and Adulthood

It is well known that ECS induces neurogenesis and by analyzing brains shortly after BrdU injection, we and others have found that proliferation is increased 2–3 days after ECS (Malberg et al. 2000; Nishida et al. 2008; Yanpallewar et al. 2012; Zhang et al. 2021). Here, by injecting BrdU 1 week prior to ECS treatment, we were able to detect enhanced survival of immature neurons independent of effects on neuronal proliferation. This result is consistent with, and extends to females, reports that extended treatment during the 4‐week immature critical period is necessary for the survival‐promoting effects of ECS (Jonckheere et al. 2018; Ueno et al. 2019). While elevated DCX^+^ cell counts after chronic ECS cannot isolate effects on proliferation versus survival of neurons born near the end of the ECS treatment, these results are consistent with other work showing that DG neurogenesis remains enhanced even in the later stages of a ~3w ECS treatment regimen (Ueno et al. 2019; Abe et al. 2024).

A recurring question is whether neurogenesis might underlie the hippocampal volume increases seen following ECT in clinical studies. Despite the robust neurogenic effects of ECS in rodent (Malberg et al. 2000; Nishida et al. 2008; Zhang et al. 2021) and primate (Perera et al. 2007) studies, it is unlikely that there is sufficient neurogenesis to account for the large‐scale volume changes seen after human ECT. In humans, ECT increases hippocampal (Nordanskog et al. 2010; Joshi et al. 2016; Oltedal et al. 2018; Argyelan et al. 2021) and DG (Takamiya, Plitman, et al. 2019; Nuninga, Mandl, Boks, et al. 2020) volume by ~5% within weeks of treatment onset. Such a large change would normally require years of neuron addition (based on human granule cell addition rates of ~2% per year (Spalding et al. 2013; Snyder 2019), and evidence that the total number of granule neurons in the rat increases but only over much longer portions of the lifespan (Ciric et al. 2026). Indeed, a recent causal examination in a mouse model found that ECS increased hippocampal volume even in irradiated mice that lacked adult neurogenesis, suggesting other trophic mechanisms are involved (Abe et al. 2024).

Nonetheless, a more complex picture arises when one considers that the DG comprised heterogeneous populations of young and old neurons that may be differentially impacted by ECS/ECT (Snyder 2019). It is typically assumed that DG neurons, once they pass through an immature critical period, will continue to survive for the lifespan. And yet, despite evidence for large cumulative effects of adult neurogenesis (~40% of neurons are adult‐born by the end of life (Snyder and Cameron 2012; Cole et al. 2020)), there are inconsistent reports on whether the granule cell population expands with age (Bayer et al. 1982; Boss et al. 1985; Rapp and Gallagher 1996). Likewise, some studies find that ECS increases total DG neuron numbers (Chen et al. 2009) and others find no change (Olesen et al. 2015). Recent work indicates that the number of DG neurons does increase over the lifespan, but net growth may only be detectable when examined over long intervals that include young adulthood, when neurogenesis rates are highest (Ciric et al. 2026). Growth is also partially offset by the loss of DBNs, which die off as a part of normal aging (Dayer et al. 2003; Cahill et al. 2017; Ciric et al. 2019, 2026). Thus, the degree to which the number of granule neurons (and hippocampal volume) increase following ECS/ECT may depend on both the rates of ABN addition and DBN loss. Here, we quantified DBNs to test the hypothesis that the large influx of ABNs promotes a homeostatic loss of DBNs. While our data do not support such a relationship, it remains possible that DBNs are culled after longer intervals (e.g., after new neurons fully integrate into the circuit over several months (Cole et al. 2020)) or that the timing of our BrdU injections labelled a population of DBNs that do not undergo cell death in adulthood (Ciric et al. 2019).

### 4.2 Morphological Plasticity in Developmentally and ABNs

Beyond the absolute numbers of cells, it is also important to consider neuronal structural changes that could alter hippocampal function. Here, we found that ECS had more dramatic effects on ABNs than DBNs. ABNs from ECS‐treated mice had longer dendrites in the ventral hippocampus, consistent with previous evidence that the ventral hippocampus shows greater volume increases following ECS in mice (Abe et al. 2024) and ECT in humans (Gyger et al. 2021). We did not observe any changes in ABN spine density, in contrast to reports that ECS increases mushroom spine density in ABNs from male MAP 6 KO mice (Jonckheere et al. 2018) and normal rats (Zhao et al. 2011). Discrepancies could be because the MAP 6 KO mouse is a model of depression and anxiety‐related behavior. Differences in the age of the cells at the time of ECS treatment could also influence plasticity responses.

Previous work has shown that ECS increases the sprouting of mossy fiber axons in rats (Vaidya et al. 1999). Here, we extend these findings by showing that ECS also increases the size of mossy fiber presynaptic terminals, specifically in ABNs and not DBNs. An important follow up will be to assess the degree to which the structural plasticity of ABNs translates to functional/physiological changes. For example, because larger MFBs are associated with larger EPSPs (Galimberti et al. 2006), our findings suggest that ECS may increase the strength of the DG‐CA3 synapse, which would have important consequences for learning and memory (Vandael and Jonas 2024). We also found that ECS‐treated males and females had more filopodia per presynaptic bouton in region CA3a. This suggests that ECS may promote new neuron activation of downstream inhibitory interneurons (Acsády et al. 1998), which could be important for DG/neurogenesis functions in sparse coding and pattern separation (Lee et al. 2015; McHugh et al. 2022).

Spine plasticity was one area where DBNs were affected by ECS. Whereas others have observed global increases in granule neuron spine density (Meyers et al. 2023), here, we found that DBNs specifically in the ventral hippocampus showed spine loss, consistent with a recent report that examined Golgi‐stained, presumably mature, DG neurons (Abe et al. 2024). This spine loss, restricted to the ventral hippocampus where ABNs showed dendritic growth, may reflect competition between ABNs and DBNs for synaptic space (McAvoy et al. 2016; Adlaf et al. 2017). Alternatively, because ECS is known to alter DG gene expression (Santiago et al. 2026) and increase reactive microglia in other areas of the hippocampus (Abe et al. 2024), other mechanisms could be involved. DBNs also displayed the addition of mushroom spines but in the dorsal, rather than ventral, DG. This differential plasticity across the longitudinal axis of the hippocampus is consistent with patterns of structural plasticity in humans (Gyger et al. 2021) and suggests that ECS may uniquely impact the memory and mood‐related functions of the hippocampus that vary along its long axis (de Hoz et al. 2003; Kheirbek et al. 2013; McNaughton and Bannerman 2024). While we did not observe any trend for dendritic plasticity in DBNs, a limitation of this experiment is that sample sizes were relatively small for some groups, due to the dense labelling that made it difficult to identify isolated cells that permitted unambiguous tracing of the full dendritic tree.

### 4.3 Comparison With Transcranial Magnetic Stimulation

It is worth considering our results in light of transcranial magnetic stimulation (TMS), another form of neurostimulation that induces neuroplasticity but is associated with fewer side effects. In contrast to ECS, TMS typically does not increase neurogenesis (Czéh et al. 2002; Cambiaghi et al. 2020; Cambiaghi et al. 2020; Zhang et al. 2021) (but see (Ueyama et al. 2011)). With respect to dendrites, TMS increases the complexity of Golgi‐stained DG granule neurons (which is likely to largely reflect DBNs and other mature neurons) (Cambiaghi et al. 2020). Using an experimental design that closely parallels the current study, we previously found that chronic theta‐patterned TMS does not affect neurogenesis and does not impact dendritic or spine structure in ABNs. However, as observed here following ECS, TMS did increase the size of MFBs and it increased numbers of bouton‐associated filopodia, but only in males (Zhang et al. 2023). Thus, growth of ABN presynaptic terminals may be a common effect of both forms of neurostimulation, where ECS has more robust effects across the sexes. That said, while our ANOVA did not reveal a treatment × sex × subregion interaction, visual inspection suggests that the trophic effects of ECS on MFBs may be limited to males in the dorsal DG (Figure 3B). Collectively, the data suggest that ECS and TMS have partially overlapping effects on DG plasticity but that ECS effects are more robust.

### 4.4 Conclusions and Future Directions

Here, we find that ECS profoundly increases neurogenesis but also promotes the growth of newborn neurons, potentially accelerating their development as is seen in preclinical models of pathological seizure (Overstreet‐Wadiche, Bromberg, et al. 2006). Consistent with a large body of work, immature ABNs were more plastic than DBNs, which were numerically stable and showed relatively modest changes in spine density. A caveat of this study is that we only looked at one time point post‐ECS, raising the question of whether differential plasticity might be observed at later timepoints. For example, because ABNs and DBNs appear to compete for synaptic space (Toni et al. 2007, 2008; McAvoy et al. 2016; Adlaf et al. 2017), it is possible that with further integration of the ECS‐induced cohort of ABNs, there is compensatory atrophy and loss of DBNs (as opposed to the relative stability/lack of plasticity that we observed). Finally, it will also be important for future studies to test whether ABNs play a preferential role in hippocampal contributions to the therapeutic and off‐target effects of ECS, so that the efficacy and specificity of ECT can be improved.

## Author Contributions

T.R.Z., F.V.R., J.S.S.: designed the study. F.V.R., J.S.S.: acquired the funding. T.R.Z., B.A., R.S., L.M.: performed the experiments. T.R.Z., J.S.S.: analyzed the data. T.R.Z., F.V.R., J.S.S.: wrote the manuscript.

## Conflicts of Interest

F.V.R. has received in‐kind equipment support for investigator‐initiated trial from MagVenture. F.V.R. has received honoraria for participation in an advisory board for Allergan. F.V.R. is a volunteer director on the board of directors of the British Columbia Schizophrenia Society. F.V.R. is a member of the Educational Committee of the Clinical TMS Society (unpaid).

## Supporting information


**Data S1:** Supporting Information.

## Data Availability

The data that support the findings of this study are available in the Supporting Information of this article.
